# Recent Progress on Hyperbranched Polymers Synthesized via Radical-Based Self-Condensing Vinyl Polymerization

**DOI:** 10.3390/polym9060188

**Published:** 2017-05-24

**Authors:** Xiaofeng Wang, Haifeng Gao

**Affiliations:** Department of Chemistry and Biochemistry, University of Notre Dame, Notre Dame, IN 46556, USA; xwang26@nd.edu

**Keywords:** hyperbranched polymer, controlled radical polymerization, inimer, transmer, self-condensing vinyl polymerization

## Abstract

This short review article summarizes recent reports on using controlled radical polymerization (CRP) of inimers (compounds containing initiating group and vinyl group in one molecule) or transmers (compounds containing chain transfer group and vinyl group in one molecule) for the synthesis of hyperbranched polymers. These inimers and transmers that carry numerous functional groups could be homopolymerized, i.e., self-condensing vinyl polymerization, or copolymerized with monovinyl monomers, i.e., self-condensing vinyl copolymerization, using atom transfer radical polymerization (ATRP), nitroxide-mediated polymerization (NMP) or reversible addition fragmentation chain transfer (RAFT) polymerization techniques, producing hyperbranched polymers and hyperstar polymers with tunable molecular weights, compositions and degree of branching. Recent reports that attempted different strategies to regulate polymer–polymer reactions were introduced, demonstrating possible syntheses of hyperbranched polymers with better defined structures and relatively low molecular weight dispersity. Finally, several CRP-produced hyperbranched polymers were discussed on their applications for encapsulation of guest molecules, nanomedicine, diagnostic imaging and catalysis.

## 1. Introduction

Highly branched polymers that are comprised of dendrimers and hyperbranched polymers represent an intriguing class of macromolecules with compact structure, high density of branching linkers, three-dimensional globular shape and multiple chain-end groups [1,2,3,4,5,6]. They have demonstrated promising properties for a variety of applications, ranging from specialty additives, lubricants and nanomedicine to molecular catalysis [7,8,9,10]. Interestingly, dendrimers and hyperbranched polymers present a sharp contrast regarding their structural controllability and synthetic simplicity. As compared to dendrimers that have an elegant structure at the cost of a sophisticated multi-step reaction [2,11,12], hyperbranched polymers from the one-pot facile polymerization suffer random bimolecular reactions with no control of polymer structures [1,3,8]. 

Till now, various techniques have been developed to synthesize hyperbranched polymers, including: (1) step-growth [3,10] or chain-growth [13,14] polymerization of AB*_m_* (*m* ≥ 2) monomers where A and B represent two functional groups that can react with each other; (2) step-growth copolymerization of A*_n_* and B*_m_* monomers (*m*,*n* ≥ 2, *m* × *n* > 4) [15,16,17]; (3) chain-growth polymerization of divinyl or multivinyl crosslinkers with or without monovinyl monomers [18,19,20,21,22,23]; (4) self-condensing ring-opening polymerization (SCROP) [24,25,26]; and (5) self-condensing vinyl polymerization (SCVP) [27]. In particular, the last method requires the use of controlled polymerization methods, such as controlled radical polymerization (CRP) [28,29,30,31,32,33,34], living ionic polymerization [35,36] and group transfer polymerization [37]. 

This review article highlights the recent progress on using CRP methods to produce hyperbranched polymers via routes of SCVP and self-condensing vinyl copolymerization (SCVCP) with monovinyl monomers. Three of the following sections focus on the use of nitroxide-mediated polymerization (NMP) [38,39,40,41] and atom transfer radical polymerization (ATRP) [42,43,44,45,46,47,48,49,50,51,52,53,54,55,56,57,58,59,60,61,62,63,64,65,66,67,68] of AB* inimers (compounds containing initiator fragment B* and vinyl group A in one molecule) and reversible addition fragmentation chain transfer (RAFT) polymerization of transmers [69,70,71,72,73,74,75,76,77,78,79,80,81,82,83,84,85,86,87,88,89,90,91,92,93,94,95,96,97,98,99,100,101,102,103] (compounds containing chain-transfer group and vinyl group in one molecule) to produce various functional hyperbranched polymers. Photo-mediated radical polymerization of polymerizable “iniferter” monomers [71,104,105,106,107] is also discussed in the section of RAFT polymerization since iniferter monomers share very similar structures as transmers. In addition, recent progress on regulating the structural heterogeneity of hyperbranched polymers and on demonstrating functions of hyperbranched polymers in various applications are discussed in Section 5 and Section 6, respectively. Meanwhile, hyperbranched polymers produced via SCVP using other initiation techniques, such as cationic polymerization [27,108], anionic polymerization [109,110,111,112,113,114,115,116], group transfer polymerization [37,117,118] and ruthenium-catalyzed coordinative polymerization [119], will not be discussed in this short review article.

## 2. Synthesis of Hyperbranched Polymers Using NMP

**Figure 1:**
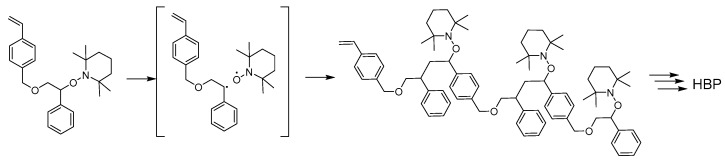
Illustration of the homopolymerization of an alkoxyamine-based inimer using nitroxide-mediated polymerization (NMP) self-condensing vinyl polymerization (SCVP) to form a hyperbranched polymer (HBP) [38].

Immediately after the first report of NMP in 1993 by Georges [120], Hawker and Fréchet quickly applied the NMP method in the first synthesis of hyperbranched polymers (Figure 1) [38]. In this case, a styrenic AB* inimer functionalized with an alkoxyamine initiating group was homopolymerized at 130 °C and produced a hyperbranched polymer in 72 h without gelation. The polymer showed an apparent molecular weight *M*_n_ = 6000 based on linear polystyrene standards with a dispersity *M*_w_/*M*_n_ = 1.40 and a glass transition temperature *T*_g_ = 45 °C. The produced hyperbranched polymer was further utilized as a macroinitiator (MI) for a second-step chain extension to produce a hyperbranched star (hyperstar) polymer with *M*_n_ = 300,000 and *M*_w_/*M*_n_ = 4.35. Cleavage of the benzyl ether linkers in the hyperbranched polymers produced degraded products with lower molecular weights, confirming the presence and nature of branching units in the hyperstar polymers. This report also represents the first example on synthesis of hyperstar polymers using hyperbranched polymers as MIs.

**Figure 2:**
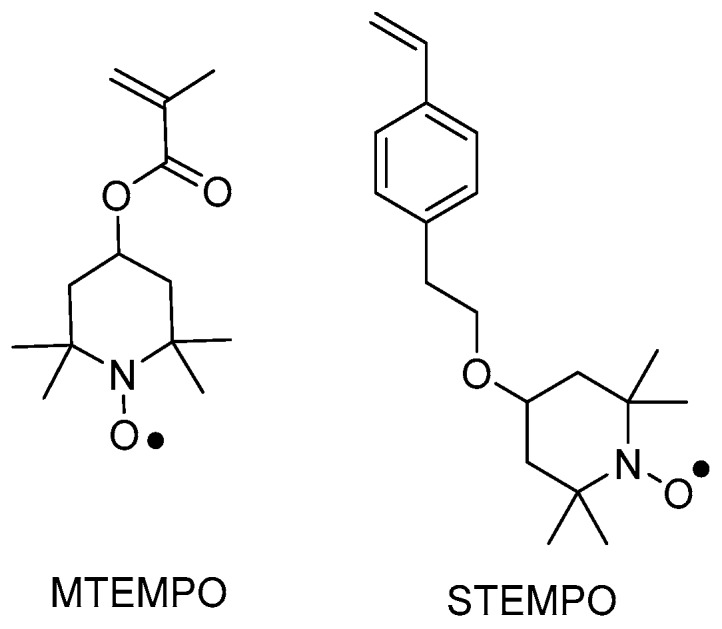
Structure of polymerizable nitroxides: 4-methacryloyloxy-2,2,6,6-tetramethyl-1-piperidinyloxy (MTEMPO) and 4-(4′-vinylphenylmethoxy)2,2,6,6-tetramethyl-1-piperidinyloxy (STEMPO).

Polymerizable nitroxides, such as 4-methacryloyloxy-2,2,6,6-tetramethyl-1-piperidinyloxy (MTEMPO) and 4-(4′-vinylphenylmethoxy)2,2,6,6-tetramethyl-1-piperidinyloxy (STEMPO) [40,72] (Figure 2), were used to introduce branching points into polymers. The branching points in these hyperbranched polymers underwent reversible thermolysis/recombination reactions at the C-nitroxide linkage, which mediates the polymerization and maintains the “livingness” of the polymers.

Overall, NMP of inimers has been applied in a couple of very first reports for the preparation of hyperbranched polymers, although its broad application is limited by some challenges, including the slow polymerization kinetics, the often required high temperature, the inability to easily control methacrylate polymerization and the multi-step synthesis of alkoxyamine-based inimers [121]. In contrast, most of the hyperbranched polymers synthesized via CRP techniques were reported using either ATRP or RAFT methods, as will be discussed in the next two sections.

## 3. Synthesis of Hyperbranched Polymers via ATRP

**Figure 3:**
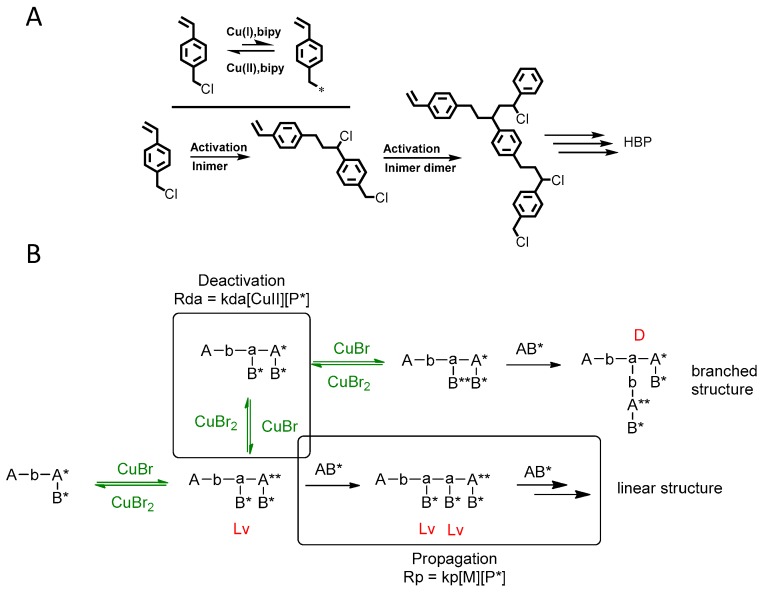
(**A**) Illustration of the homopolymerization of *p*-(chloromethyl)styrene (CMS) by atom transfer radical polymerization (ATRP) SCVP to form a hyperbranched polymer [42]; (**B**) Dynamic exchange during ATRP of inimer [47], A* and B* representing two types of dormant alkyl halogen initiating groups; A** and B** represent two types of propagating radicals.

The first ATRP SCVP of inimer was reported by Matyjaszewski using commercially available, *p*-(chloromethyl)styrene (CMS), in the presence of Cu(I) and 2,2′-bipyridine (bpy) [42] (Figure 3A). The structures of various AB* inimers reported in the ATRP SCVP and ATRP SCVCP so far are listed in Table 1. Three types of inimers in terms of polymerizable vinyl group (A group) could be found in published reports covering: (1) acrylate inimers (AB*1–9); (2) styrenyl inimers (AB*10, AB*11); and (3) methacrylate inimers (AB*12–20). The other ends of these AB* inimers all contained alkyl halide groups: either alkyl bromide or alkyl chloride, which generally employed Cu-based catalyst for initiating the polymerization. 

In general, the branched structure of hyperbranched polymers synthesized via ATRP of AB* inimers is critically affected by the competition between radical propagation and deactivation reactions. Conceptually, a propagating radical from the activation of alkyl halide could either react with a new inimer to form a linear unit (such as Lv) or be deactivated by reacting with Cu(II) deactivator (Figure 3B). An increased rate ratio of propagation over deactivation would produce more Lv linear units from one radical in an activation/deactivation cycle and lower the degree of branching (DB) of polymers. In contrast, a fast deactivation reaction could quickly stop the propagation of linear units and raise the chance of activating a different alkyl halide in another activation/deactivation cycle, which is the essential step to form a branched unit (D in Figure 3B) [47,67,68,122]. 

It is useful to note that SCVP of inimers has both step-growth and chain-growth mechanistic features; high molecular weights and highly branched polymer structures could only be achieved at very high conversion. It was reported that SCVP of inimers AB*13 and AB*14 could not produce high molecular weight polymers using various ligands and temperatures mainly because of the fast radical termination reaction forming an excess amount of deactivator Cu(II) species. Thus, Cu-based ATRP with the addition of Cu(0) for AB*13 and AB*14 [123] and Ni-based CRP for AB*13 [52] were applied to achieve better results. The accumulated deactivator Cu(II) during ATRP of inimers could also be removed using a heterogeneous microemulsion polymerization system. For instance, the polymerization of inimer AB*13 in a microemulsion not only regulated the polymer structure based on the dimension and uniformity of the discrete latexes, but also significantly increased the polymerization kinetics via the effective partition of Cu(II) deactivator into aqueous media [45,47]. 

**Figure 4:**
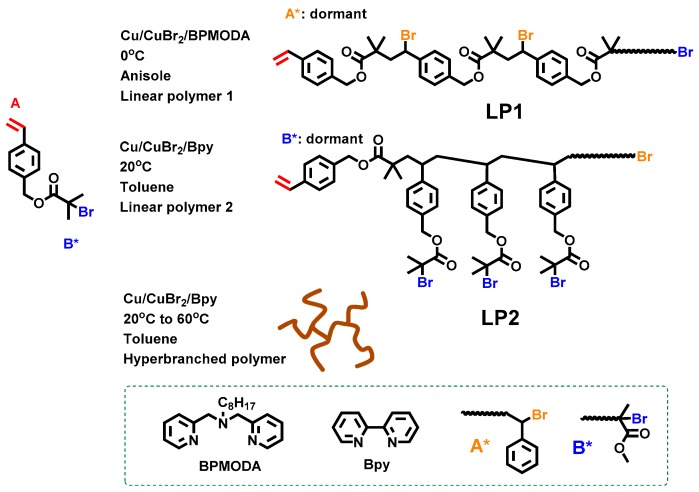
Control of polymer architectures in the ATRP SCVP of AB*10. Reproduced with permission [65], copyright 2010 American Chemical Society

So far, a few reports have been published on ATRP SCVP of styrenic AB* inimers. AB*10 and AB*11 as shown in Table 1 are two representative examples, in which the asymmetric structure of AB*10 offered tunable structures from linear to hyperbranched under different temperatures and ligands [65]. Through manipulating three polymerization parameters including temperature, ligand and solvents, the authors could manipulate the reactivities of initiating groups and catalysts and the solubility of deactivators in the polymerization solution, resulting in polymers with different architectures [67,68]. Low reaction temperature with high concentration of Cu(II) deactivator gave a higher chance of activation from the formed A* initiating site, forming linear polyester LP1. In contrast, the use of less reactive and low-concentration deactivator promoted the polymerization from B* sites and produced linear polymer LP2 (Figure 4). Between these two situations, a series of branched polymers with different DB values could be produced by simply tuning the reaction temperature and the effective concentration of deactivators in solution. The linear polymers LP1 and LP2 that carried many reactive benzylic bromides on the backbone have further potentials for modification via organic and polymerization reactions to incorporate additional functionalities on the polymers.

## 4. Synthesis of Hyperbranched Polymers by RAFT

**Figure 5:**
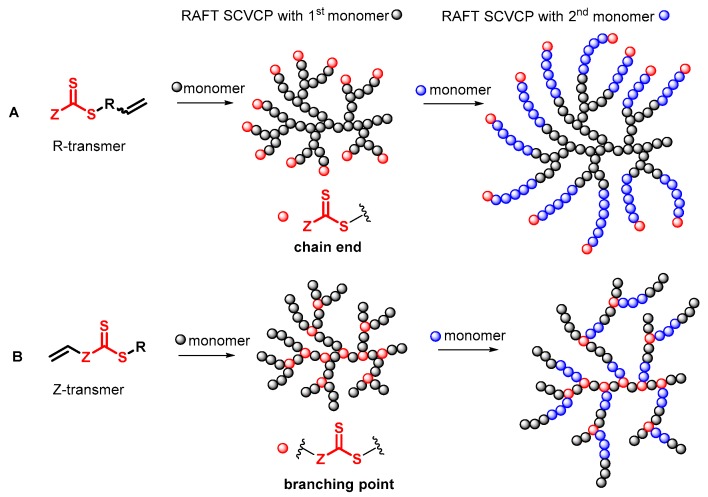
General structures of transmers for (**A**) R method and (**B**) Z method to produce hyperbranched polymers.

Chain transfer agent (CTA), usually a thiocarbonylthio compound with a structure as Z(C=S)SR, is the key component in the RAFT process, which mediates the equilibrium between active and dormant chains. The Z-group activates the thiocarbonyl double bond and provides stability to the intermediate adduct formed when radicals are added to the CTA, while the R-group is a good leaving group capable of reinitiating RAFT polymerization after fragmentation. Similar as an AB* inimer in ATRP and NMP, the monomer used in RAFT SCVP composes a polymerizable vinyl group (A group) installed into the CTA structure (equivalent to the B* group) on either the R-group or Z-group side. Different from the AB* inimer that has a real initiating group B*, the polymerizable CTA used in the RAFT method needs an external radical source to cleave the S–R bond and generate a propagating radical. This structural difference gives the polymerizable CTA another name, i.e., transmer. Although designing a transmer by placing a polymerizable vinyl group at either the R group (R approach) or the Z group (Z approach) is available, two main limitations of the Z-approach become significant: steric hindrance to access the CTA functionalities and the potential weakness of the branch points (Figure 5) [71,128]. To solve this problem, Puskas and coworkers reported the first bulk polymerization of styrene with 4-vinylbenzyl dithiobenzoate, which avoided introducing the CTA into the branching point (R method; Figure 5A) [78]. Till now, the R method was utilized more frequently to produce a variety of branched polymer structures. Table 2 summarizes the transmers that have been prepared and reported to date. While the first transmer reported was strictly limited to styrenic polymerizable group, new examples of transmers with acrylate/methacrylate polymerizable groups were developed [77]. Xanthate-based transmers reported by both Poly’s [81] and Zhou’s [83] groups were subsequently used to prepare hyperbranched poly(vinyl acetate). The development of these new transmers was crucial for this vinyl ester monomer because poor control was always achieved using any other CTAs. The most commonly reported iniferter monomers contain dithiocarbamate (DC) chain-transfer group. Photopolymerizations in benzene solution of 2-(*N,N*-diethylaminodithiocarbamoyl)ethyl methacrylate (DTCM, Entry 21 Table 2) were carried out by Mori et al. [106] via irradiation with UV light in a sealed glass ampoule under high vacuum at 20 °C.

In the RAFT polymerization of transmers, so far, only a handful of examples have reported the homopolymerization of transmers. In 2011, Zhao and coworkers [82] used *S*-(4-vinyl)benzyl *S*’-propyl trithiocarbonate (VBPT) to prepare a hyperbranched polymer as a control experiment with the intention to compare the solution behavior of branched copolymers obtained by RAFT SCVCP. A thorough literature research to the best of our knowledge indicates that RAFT homopolymerization of transmers when using thermal initiators as radical sources could only produce low molecular weight hyperbranched polymers. The highest molecular weights available in several reports include *M*_n_ = 24,700 in Zhao’s group [82], *M*_n_ = 8740 in Sumerlin’s group [80] and *M*_n_ < 1000 in Poly’s group [81]. On the other hand, Ishizu and Tanaka reported the photo-polymerization of iniferter monomers and produced hyperbranched polymers with molecular weight above 100,000 [129,130]. Recently, our group reported a systematic study of the homopolymerization of a trithiocarbonate transmer and produced the first hyperbranched polymers with over half-a-million molecular weight by applying a concurrent ATRP/RAFT initiation process that generated radicals without the use of thermal initiator [131]. 

Most of the RAFT polymerizations of transmers were conducted by SCVCP with various functional monovinyl monomers, which makes it easy to tune the DB and introduce reactive groups in the segmented hyperbranched polymers (SHBPs). The addition of monovinyl monomers diluted the branching density and consequently lowered the DB in general. To achieve a uniform distribution of branching units in the copolymer structure, similar reactivity between the polymerizable group of the transmer and the vinyl group of the comonomer is critical. The first report of the RAFT SCVCP was published in 2003 by Yang et al., who utilized RAFT SCVCP to prepare hyperbranched polystyrene [72]. The transmer structure was based on a styrenic dithioester transmer 1 (Table 2) with a styrenyl unit as the Z-group. By introducing the dithioester group into the branching point, it offers an advantage to prepare segmented hyperbranched copolymers through a two-step polymerization method. As shown in Figure 5B, one monovinyl monomer was copolymerized with transmer first to produce a hyperbranched macro-transfer agent before the polymerization of a second monomer by inserting the second block at the reactive CTA branching points, generating branched block-copolymers. In contrast, star-shaped copolymers would be produced if the hyperbranched polymers were made out of the R method since the CTA groups are at the periphery of the hyperbranched polymers (Figure 5A). This was utilized by Patrickios and coworkers to produce segmented amphiphilic hyperbranched polymers of styrene and vinylpyridine through stepwise RAFT SCVP [97]. In comparison to NMP SCVP and ATRP SCVP, which could only provide star-shaped copolymers, RAFT SCVP and RAFT SCVCP offer more options to alter the polymer structures by using different transmer structures.

## 5. Attempts to Regulate the Structures of Hyperbranched Polymers

Most of the reported hyperbranched polymers were synthesized by radical polymerization in bulk or solution. The homogeneous reaction media cause random monomer–monomer, monomer–polymer and polymer–polymer reactions throughout the reactor, which leads to polymers with poorly defined structure, i.e., high dispersity. Since the physical properties of hyperbranched polymers are critically influenced by their molecular weights and structural uniformity, it is highly desirable to develop robust synthetic methods that can regulate the molecular weight distribution (MWD) of hyperbranched polymers with minimal compromise of the facile one-pot synthesis feature. So far, two major methods were applied to provide a better control over the dispersity of hyperbranched polymers. The first method is the application of a multifunctional core molecule via either slow addition of monomers into a dilute solution of the multifunctional core or the use of core molecule carrying more reactive groups than those on monomers. The main concept is to achieve a desired chain-growth polymerization via selective monomer-polymer reactions and disfavor monomer-monomer reactions. In contrast, the second method focuses on segregating the polymerization of monomers in a confined nanospace in order to decrease the dispersity of hyperbranched polymers.

**Figure 6:**
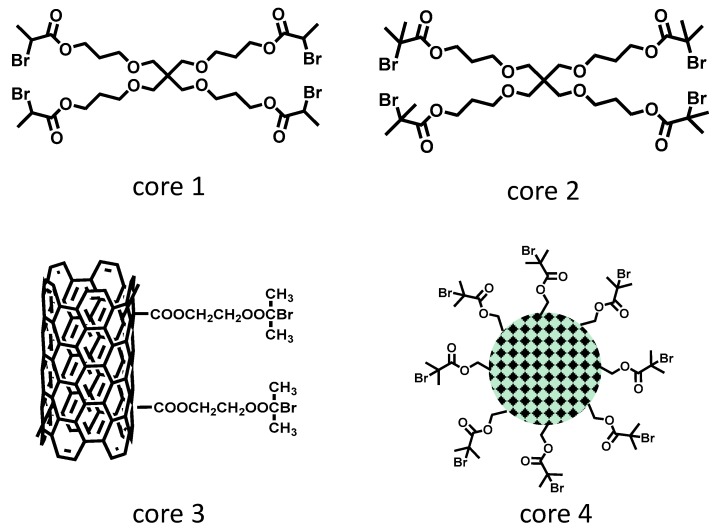
Structure of multifunctional cores reported in recent publications to produce hyperbranched polymers with a narrow molecular weight distribution (MWD).

According to the simulation reported by Frey et al. [133] and Müller et al. [134], the slow addition of AB*_m_* monomers or AB* inimers into a solution of the multifunctional B*_x_* (*x* ≥ 2) core could decrease the dispersity and increase the DB value of the hyperbranched polymers. In a situation when AB*_m_* monomers only react with the B functional groups on the B*_x_* core, at complete conversion, hyperbranched polymers with *M*_w_/*M*_n_ = 1 + (*m* − 1)/*x* are produced. Based on this theory, many research groups applied a variety of AB*_m_* + B*_x_* pairs and achieved experimental results qualitatively supporting the simulation. However, examples of radical polymerization using AB* inimer and B_x_* core are rare. Pan et al. applied a tetrafunctional initiator core (Core 1, Figure 6) in the ATRP SCVP of inimer (AB*15, Table 1) and produced hyperbranched polymers with the most uniform structure (*M*_w_/*M*_n_ = 2.16) with a molar ratio of [AB*15]_0_/[core 1]_0_ = 28 [51]. In order to obtain hyperbranched polymers with even lower dispersity (e.g., *M*_w_/*M*_n_ = 1.5), the initial feed ratio of inimer to core was limited to less than 100:1, and the polymerization required slow monomer addition during the polymerization. Different from using the slow monomer addition strategy to favor the monomer–polymer reaction, increasing the reactivity of the core molecules with respect to monomers could also decrease the dispersity of the resulting polymers. The strategy of using a high-reactivity core (Core 2, Figure 6) was applied in the ATRP SCVP of inimers AB*8 (Table 1) to synthesize hyperbranched polymers with low dispersity [50]. Within the studies, an optimal feed ratio of [AB*8]_0_/[Core 2]_0_ = 40:1 in one pot produced polymers with *M*_w_/*M*_n_ < 1.5.

As an alternative method, the growth of hyperbranched polymers from an insoluble support could also lower the polymer dispersity. Moore et al. applied a multifunctional ATRP initiator based on surface-functionalized multi-walled carbon nanotubes (Core 3, Figure 6) for the polymerization of AB*8 (Table 1) [135]. The resulting nanotubes with a hyperbranched polymer shell showed good dispersibility in THF and CHCl_3_, although no DB and dispersity values were determined. A second example synthesized hyperbranched polymers grafted on the exterior surface of mesoporous silica nanoparticles (MSN, Core 4, Figure 6) by surface-initiated ATRP of AB*8 (Table 1) [59]. It was found that the molecular weights of hyperbranched polymers after cleavage from the MSN core increased from *M*_n_ = 18.6 × 10^3^–29.2 × 10^3^ when the initial weight ratios of [AB*8]_0_/[Core 4]_0_ changed from 30–125 with relatively low dispersity (*M*_w_/*M*_n_ = 1.80–2.30).

**Figure 7:**
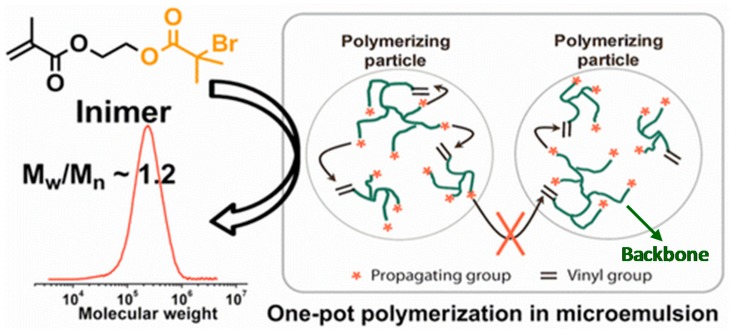
Polymerization of inimers in microemulsion. Reproduced with permission [45], copyright 2012 American Chemical Society*.*

In contrast to the homogeneous solution polymerization, our group recently reported the application of heterogeneous micelle-based confined space, i.e., microemulsion, to regulate the synthesis of hyperbranched polymers by conducting one-pot ATRP SCVP of AB* inimers (Figure 7) [45]. The random polymer–polymer reactions were effectively confined in each discrete polymerizing nanoparticle in the microemulsion, and the obtained hyperbranched polymers showed narrow MWD and a hydrodynamic size similar to that of nanoparticles, i.e., one hyperbranched polymer per latex particle by the end of the polymerization. This ATRP of inimers in heterogeneous microemulsion has been demonstrated as a robust method by exploring five methacrylate-based inimers under various microemulsion conditions [47]. The produced hyperbranched polymers showed varied compositions with tunable DB = 0.26–0.41, molecular weights (*M*_n_ = 194 × 10^3^–1301 × 10^3^) and low dispersity (*M*_w_/*M*_n_ = 1.1–1.7).

Meanwhile, all hyperbranched polymers produced within the spatially-confined micelles (i.e., latexes) contained hundreds of bromine initiating sites. They could be subsequently used as MIs for polymerization of another functional monomer to synthesize hyperstar polymers with core-shell structures. The composition and dimension of the core and shell segments could be independently controlled based on several experimental variables, including the monomer species, the feed ratios and the conversions. Very recently, this type of synthesis was further accomplished in a one-pot procedure via sequential polymerization of AB*_m_* monomer and a second functional monomer in the oil-in-water emulsion. The surfactant-protected emulsion latexes functioned as segregated reactors to effectively eliminate the undesired star-star coupling, achieving both high monomer conversion and fast polymerization at the same time [48]. We anticipate that the general concept of polymerization in confined space will enable many synthetic opportunities to better regulate the side reactions and produce various structurally-defined polymer nanostructures, including star polymers, cyclic polymers and graft polymers. In addition, Jiang’s group conducted a radical copolymerization of styrene with Transmer 23 (Table 2) (3-mercaptohexyl methacrylate) in an aqueous emulsion system with high monomer/transmer ratio (100/25), producing branched polymers with *M*_w_/*M*_n_ = 5.37 [132]. As compared to the previous microemulsion system, this emulsion polymerization seems less effective in controlling the polymer structure, probably due to the poor uniformity of the emulsion latexes and the presence of many branched polymers per latex particle.

## 6. Applications of Hyperbranched Polymers Produced from Radical-Based SCVP

In general, hyperbranched polymers display many useful properties, such as highly compact structure, few chain entanglements, high solubility, low viscosity and multiple peripheral groups. They have been utilized in various fields ranging from photoelectric materials, nanotechnology, biomedicines, composites, coatings, adhesives and lubricants. Regarding the hyperbranched polymers produced from controlled radical SCVP, a few examples have been published recently giving emphasis on the functionality.

### 6.1. Hyperbranched Polymers for Loading Guest Molecules

**Figure 8:**
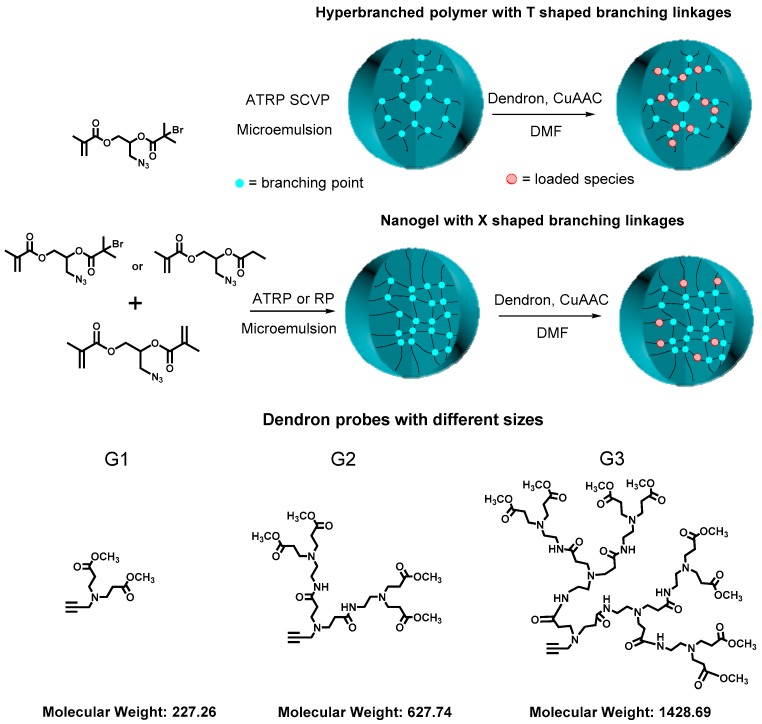
Structures of hyperbranched polymers and crosslinked nanogels and their influence on loading efficiency of Dendron molecules. Reproduced with permission [49], copyright 2015 John Wiley and Sons.

Hyperbranched polymers are considered as unimolecular containers and could be applied as hosts to encapsulate small guest molecules. The loading capability of hyperbranched polymers is one of the most important parameters, which is critically affected by the density and functionality of the branching points. For instance, as compared to commonly-used nanogels that have an “X”-shaped branching unit from which four chains radiate out, hyperbranched polymers have a “T”-shaped branching unit from which three arms radiate out (Figure 8). The effect of branching unit structure on the loading properties of these two unimolecular containers was first studied by our group. A family of hyperbranched polymers and crosslinked nanogels with similar molecular weights, similar hydrodynamic diameters in THF and the same density of azido groups per structural unit were synthesized via ATRP and conventional radical polymerization (RP) of azido-functionalized inimers, divinyl cross-linkers and monovinyl monomers in microemulsion. The hyperbranched polymers demonstrated three-times higher loading efficiency than crosslinked nanogels when both reacted with three alkynyl-containing dendron molecules using Cu-catalyzed azide-alkyne cycloaddition (CuAAC) reactions (Figure 8) [49]. 

In a separate study, the Gao Chao group at Zhejiang University reported that the high polarity difference between the core and shell of an amphiphilic hyperbranched polymer could selectively load organic dyes into the core domain [92]. In this study, core–shell amphiphilic hyperbranched polymers were produced by RAFT SCVCP of Transmer 9 (Table 2) and 2-(dimethylamino)ethyl methacrylate (DMAEMA) monomer in one pot. After chain extension with polystyrene to introduce the hydrophobic shell, the multifunctional tertiary amino groups in the core were converted into quaternary ammonium by reacting with propargyl bromide, which can efficiently load azido-functionalized dye molecules into the charged hydrophilic core area in high efficiency.

### 6.2. Application of Hyperbranched Polymers for Drug Delivery

**Figure 9:**
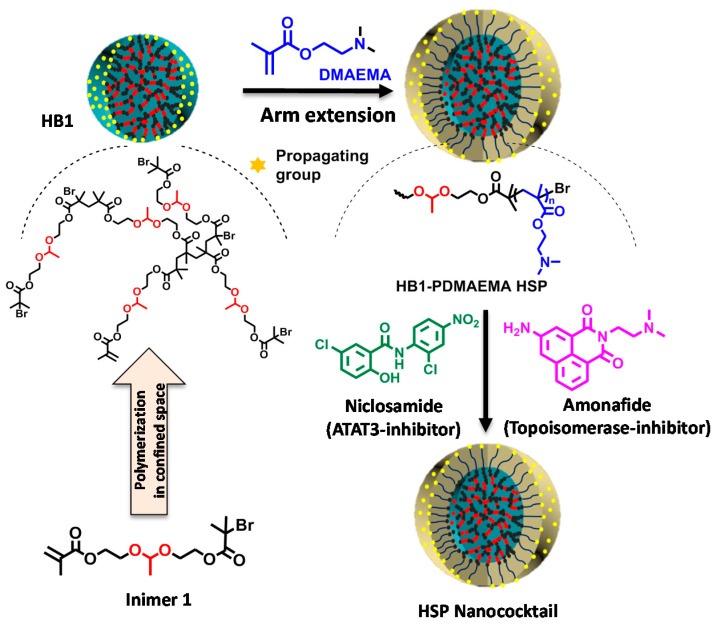
Hyperbranched macroinitiators (MIs) for the synthesis of degradable hyperstar polymers (HSPs) in water. Reproduced with permission [136], copyright 2015 Royal Society of Chemistry*.* DMAEMA, 2-(dimethylamino)ethyl methacrylate.

Hyperbranched polymers have potential applications in nanomedicine because of their compact architecture and multiple peripheral chain ends, enhancing multivalent targeting efficiency. In 2015, Pan’s and Gao’s groups reported the ability of a novel combinatorial therapy targeting triple negative breast cancer cells using a hyperstar polymer (HSP) to encapsulate two enzyme inhibitors inside: niclosamide (e.g., 5-chloro-*N*-(2-chloro-4-nitrophenyl)-2-hydroxybenzamide), a known STAT3 inhibitor and amonafide (e.g., 5-amino-2-[2-(dimethylamino)ethyl]-1H-benzo[de]isoquinoline-1,3(2H)-dione), an agent activating topoisomerase-II pathways [136]. The HSP was constructed in two steps: ATRP of AB*19 inimer (Table 1) in microemulsion followed by a surface-initiated ATRP of DMAEMA in solution (Figure 9). After encapsulation of given amounts of inhibitors, this nano-cocktail showed at least 2–4-fold better inhibition of triple negative breast cancer cells than the individual drugs and 6–20-times more selective than the parent drugs.

**Figure 10:**
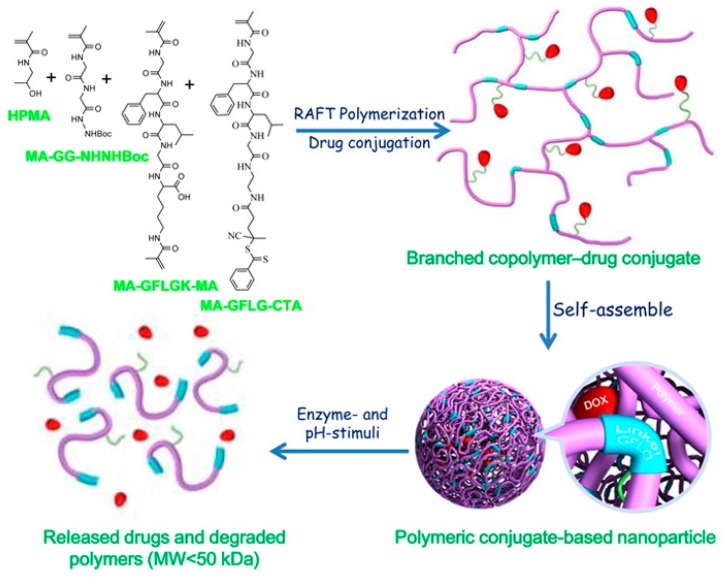
Schematic representation of the synthesis, assembly and degradation of poly[*N*-(2-hydroxypropyl)methacrylamide] (polyHPMA) copolymer. Reproduced with permission [137], copyright 2016 American Chemical Society*.*

Gong et al. reported an enzyme- and pH-sensitive branched poly[*N*-(2-hydroxypropyl)methacrylamide] (polyHPMA) copolymer-doxorubicin conjugate, which was prepared via a one-pot RAFT SCVCP of one transmer and three functional monomers followed by drug conjugation (Figure 10) [137]. The amphiphilic polymer–drug conjugates formed nanoparticles in water, but degraded to low molecular weight products in the presence of papain or cathepsin B. The drug–conjugated polymer nanoparticles exhibited greater accumulation in breast tumors and released doxorubicin molecules under acidic environment, resulting in enhanced antitumor therapeutic indexes with targeted delivery.

**Figure 11:**
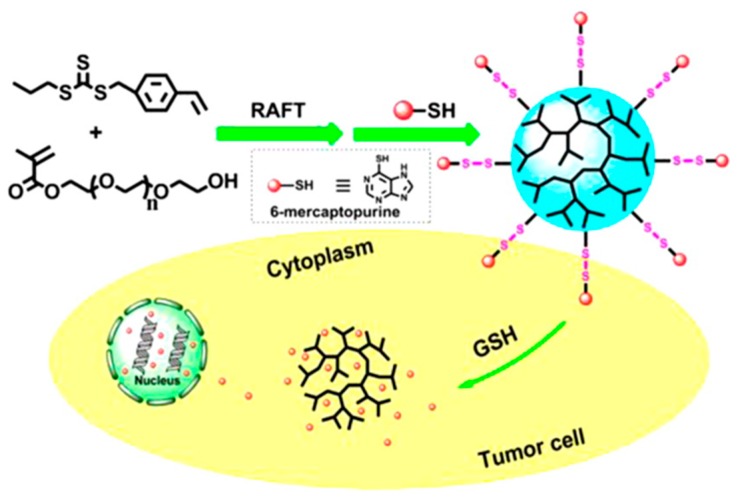
Redox-responsive branched polymer-drug conjugate with branched poly(*S*′-propyl trithiocarbonate (VBPT)-*co*-PEGMA). Reproduced with permission [93], copyright 2014 American Chemical Society*.*

In another report, Lu and coworkers prepared a redox-responsive branched polymer-drug conjugate, in which the branched poly(VBPT-*co*-PEGMA) was synthesized by one-pot RAFT copolymerization of *S*-(4-vinyl) benzyl *S*′-propyl trithiocarbonate (VBPT) transmer and poly(ethylene glycol) methacrylate (PEGMA) monomer followed by several steps of chain-end transformations (Figure 11) [93]. The amphiphilic polymer–drug conjugates self-assembled into micelles in aqueous solution and destructed under the reductive cellular environment to release the anticancer drug 6-mercaptopurine.

### 6.3. Application of Hyperbranched Polymers for Bioimaging

**Figure 12:**
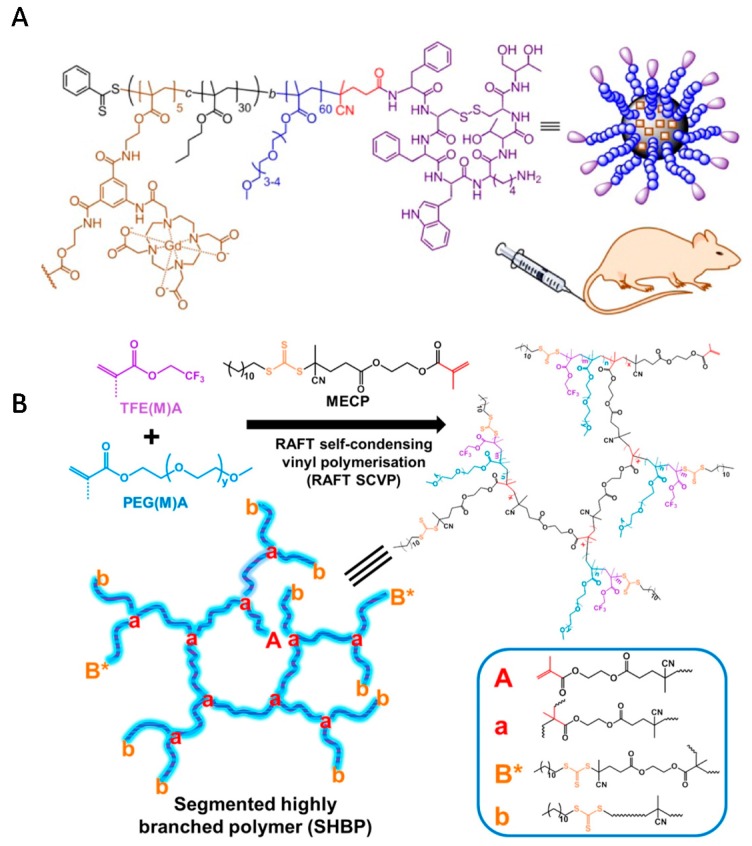
(**A**) Octreotide-functionalized contrast agent for targeted MRI; (**B**) illustration of the synthesis of segmented hyperbranched polymers via RAFT SCVP. Reproduced with permission [98,139], copyright 2015 and 2016 American Chemical Society.

There is great interest in developing active targeting nanomaterials for various diagnostic bioimaging applications, such as magnetic resonance imaging (MRI), to alter biodistribution and improve imaging sensitivity [138]. Robins et al. used RAFT SCVP and synthesized branched block copolymers carrying 1,4,7,10-tetraazacyclododecane-*N,N′,N″,N‴*-tetraacetic acid (DO3A) macrocycles within their cores and octreotide (somatostatin mimic) cyclic peptides at their periphery (Figure 12A) [139]. These polymeric nanoparticles have been chelated with Gd^3+^ and applied as MRI nanocontrast agents. As another example of the application of hyperbranched polymer in MRI, Whittaker et al. produced a series of highly branched polymers consisting of fluoro- and PEG-based segments by RAFT SCVCP (Figure 12B) [98]. It was demonstrated that the chain sequence of fluorinated monomers along the polymer backbone and the polymer DB values significantly determined the final ^19^F NMR properties. 

### 6.4. Application of Hyperbranched Polymers for Catalysis

**Figure 13:**
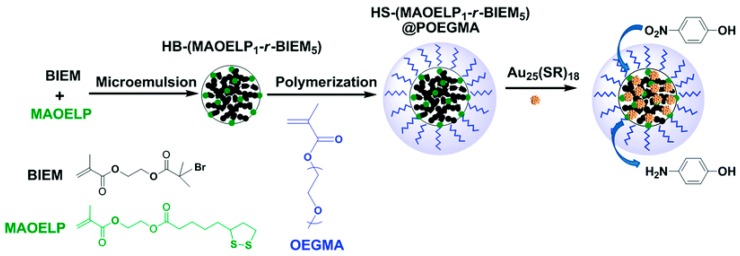
Preparation of HS–(MAOELP_1_-r-BIEM_5_)@POEGMA stabilized Au_25_(SR)_18_ for catalytic reduction of 4-nitrophenol. Reproduced with permission [140], copyright 2017 Royal Society of Chemistry*.* OEGMA, oligo(ethylene glycol) methyl ether methacrylate.

Hyperbranched polymers synthesized via CRP methods have also been used as unimolecular containers to stabilize catalytic metal nanoparticles inside. Recently, our group developed a type of reusable hyperstar polymer–Au_25_(SR)_18_ nanocomposites for catalysis of reduction of 4-nitrophenol by NaBH_4_ [140]. A hyperbranched copolymer was first constructed via ATRP SCVCP of AB*13 inimer (shown as BIEM in Figure 13) with a cyclic disulfide-containing methacrylate monomer (MAOELP) in microemulsion in a 5:1 molar feed ratio, before it was used as an MI for polymerization of oligo(ethylene glycol) methyl ether methacrylate (OEGMA, *M*_n_ = 500) to grow the radiating arms. The hyperstar polymer HS–(MAOELP_1_-r-BIEM_5_)@POEGMA with disulfide groups was proven to efficiently encapsulate Au_25_(SR)_18_ nanoclusters through ligand exchange without destroying the fine structure of the Au_25_(SR)_18_ clusters. The obtained hyperstar–Au_25_(SR)_18_ nanocomposites showed great stability with no size change after a three-month shelf storage. They were used as efficient catalysts for the catalytic reduction of 4-nitrophenol by NaBH_4_ in five cycles, showing convenient catalyst recovery without losing catalytic efficiency.

## 7. Conclusions and Outlook

Here, we summarize the recent progress on using CRP methods, including NMP, ATRP and RAFT, to produce hyperbranched polymers and their exploration as materials in numerous applications. By copolymerizing inimers or transmers with monovinyl monomers, the SCVP technique could be extended to SCVCP that allows easy functionalization of the hyperbranched copolymers with various reactive groups and tunable DB values. Currently, most of the hyperbranched polymers synthesized by using this method suffered from limited structural control, i.e., the broad molecular weight distribution, due to the random polymer-polymer reaction. Several methods have been explored to better control the polymer structures, such as the application of a multifunctional core and the use of a confined space. Robust synthetic methods are still in high demand that can achieve accurate placement of one or a few monomers per time into a hyperbranched polymer with the orthogonality of reactive groups for the modification in different domains. In particular, photo-mediated CRP methods [141,142], any ATRP techniques with ppm amount of Cu [143] or no Cu catalysts [144,145] are expected to be applied in the polymerization of inimers and transmers for producing hyperbranched polymers with robust conditions, free of catalyst contaminants and better control of polymer structures [146]. Meanwhile, the intriguing features of these hyperbranched polymers for easy synthesis and multiple chain-end groups have attracted the exploration of their applications as materials in drug delivery, imaging diagnostics and catalysis. These progresses establish a more comprehensive structure-property relationship about these CRP-produced hyperbranched polymers for their broad utilization in industrial applications.

## Figures and Tables

**Table 1:** Summary of AB* inimers used in ATRP SCVP.

AB* Reference	Structure	AB* Reference	Structure
AB*1 [43,122]	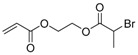	AB*11 [58]	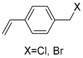
AB*2 [123]	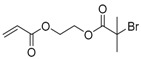	AB*12 [47,48]	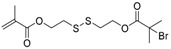
AB*3 [124]	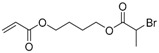	AB*13 [45,52]	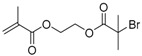
AB*4 [55]	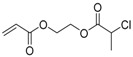	AB*14 [123]	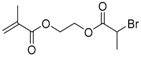
AB*5 [46]	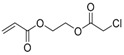	AB*15 [50]	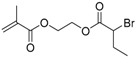
AB*6 [125]	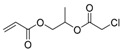	AB*16 [56]	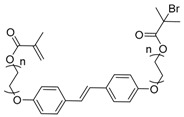
AB*7 [44]	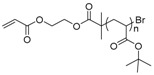	AB*17 [126]	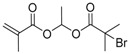
AB*8 [50]	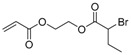	AB*18 [47,127]	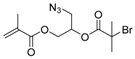
AB*9 [60]	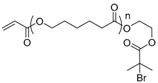	AB*19 [47]	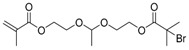
AB*10 [65]	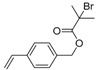	AB*20 [47]	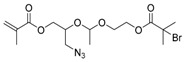

**Table 2:** Summary of transmers used in RAFT SCVP.

Transmer	Structure	Vinyl Group ^a^	CT Group ^b^	Reference
1	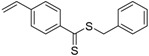	S	DTB	[72,78,86,97]
2	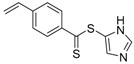	S	DTB	[73,74,76]
3	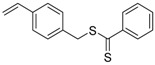	S	DTB	[89,97]
4	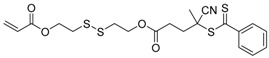	A	DTB	[96]
5	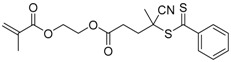	M	DTB	[85]
6	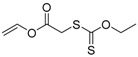	VA	DTC	[83]
7	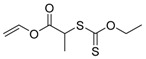	VA	DTC	[81]
8	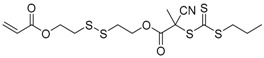	A	TTC	[102]
9	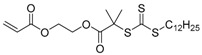	A	TTC	[84,92,101]
10	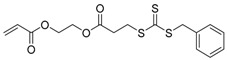	A	TTC	[85]
12	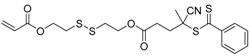	A	TTC	[88]
13	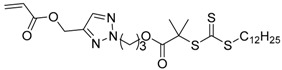	A	TTC	[77,80]
14	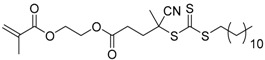	M	TTC	[98]
15	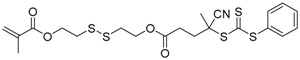	M	TTC	[94]
16	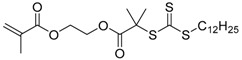	M	TTC	[87,92]
17	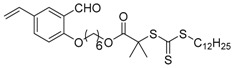	S	TTC	[95]
18	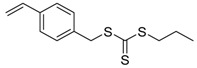	S	TTC	[82,93]
19	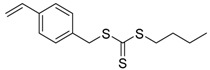	S	TTC	[89,90]
20	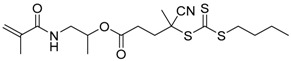	M	TTC	[99]
21	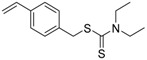	S	Carbamate/iniferter	[129]
22	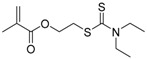	M	Carbamate/iniferter	[106]
23	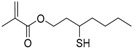	M	Thiol	[132]

^a^ Vinyl groups include: S: styrenyl; A: acrylate; M: methacrylate; VA: vinyl acetate; ^b^ chain transfer (CT) groups include: DTB: dithiobenzoate; TTC: trithiocarbonate; DTC: xanthate (dithiocarbonate); TTC: trithiocarbonate.
